# Cell-Mediated Immune Responses in Paraneoplastic Neurological Syndromes

**DOI:** 10.1155/2013/630602

**Published:** 2013-12-30

**Authors:** Mikolaj Piotr Zaborowski, Slawomir Michalak

**Affiliations:** ^1^Division of Gynecologic Oncology, Department of Gynecology, Obstetrics and Gynecologic Oncology, Poznan University of Medical Sciences, Polna Street 33, 60-535 Poznan, Poland; ^2^Department of Neurochemistry and Neuropathology, Poznan University of Medical Sciences, Przybyszewskiego Street 49, 60-355 Poznan, Poland; ^3^Neuroimmunological Unit, Polish Academy of Sciences, Przybyszewskiego Street 49, 60-355 Poznan, Poland

## Abstract

Paraneoplastic neurological syndromes (PNS) are disorders of the nervous system that are associated with remote effects of malignancy. PNS are considered to have an autoimmune pathology. It has been suggested that immune antitumor responses are the origin of improved outcome in PNS. We describe cell-mediated immune responses in PNS and their potential contributions to antitumor reactions. Experimental and neuropathological studies have revealed infiltrates in nervous tissue and disturbances in lymphocyte populations in both cerebrospinal fluid and peripheral blood. A predominance of cytotoxic T lymphocytes (CTLs) over T helper cells has been observed. CTLs can be specifically aggressive against antigens shared by tumors and nervous tissue. Based on genetic studies, a common clonal origin of lymphocytes from blood, tumor, and nervous tissue is suggested. Suppressive regulatory T (Treg) lymphocytes are dysfunctional. Simultaneously, in tumor tissue, more intense cell-mediated immune responses are observed, which often coincide with a less aggressive course of neoplastic disease. An increased titer of onconeural antibodies is also related to better prognoses in patients without PNS. The evaluation of onconeural and neuronal surface antibodies was recommended in current guidelines. The link between PNS emergence and antitumor responses may result from more active CTLs and less functional Treg lymphocytes.

## 1. Introduction

Paraneoplastic neurological syndromes (PNS) are defined as disorders of the nervous system that are due to a neoplasm but exclude tumor infiltration, compression, or metastasis [1]. The diagnostic criteria of definite PNS include the manifestation of the classical (typical) syndrome and the detection of onconeural antibodies [2] that can be associated with clinically evident malignant tumors [1]. Paraneoplastic reactions can affect both peripheral and central nervous systems. The most common syndromes and associated tumors are summarized in Table 1. Neurological syndromes frequently precede the clinical manifestation of a tumor by months [3]. It appears that, in this group of patients, the neoplasms are less advanced, metastases are less frequent, overall survival is better [4, 5], and single cases of tumor regression have been reported [6]. Such clinical observations suggest a naturally occurring antitumor immune response in PNS patients [7]. PNS are regarded as autoimmune disorders. In this review, we focus on the cell-mediated immune responses in the course of PNS and neoplastic disease in order to show the potential points of interplay between them that may have impact on tumor progression.

## 2. Cell-Mediated Responses in PNS

The prevailing view on the pathogenesis of PNS is that tumor cells share antigens with nervous tissue. As a result, an immune response that is directed against the neoplasm cross-reacts with neurons. These shared antigens are referred to as onconeural antigens, whereas antibodies against them are also known as onconeural. The most common onconeural antibodies and associated tumors are presented in Table 2. A detection of onconeural antibodies plays a key role in the PNS diagnosis [1]. However, the involvement of the humoral response in the pathogenic mechanism remains unclear. Studies on antibody transfer to animals have been successful in inducing Lambert-Eaton myasthenic syndrome [8] and cerebellar syndromes [9, 10]. The *in vitro* neurotoxicity of anti-Hu [11] and anti-Yo antibodies [12] has been shown. Pathological studies have revealed the presence of IgG deposits around neurons in dorsal root ganglia in patients affected by paraneoplastic encephalomyelitis (PEM) that is associated with anti-Hu antibodies [13]. IgG deposites have also been identified in the cytoplasm and nuclei of neurons of the dorsal root ganglia in the course of anti-Hu-positive paraneoplastic subacute 4 sensory neuronopathy [14]. This finding has been corroborated by the detection of the anti-Hu antibodies in the nuclei of neurons in the central nervous system in patients with PEM/sensory neuronopathy syndrome [15]. All of the abovementioned reports have focused on the immune responses against intracellular antigens.

Recently, neuronal surface antibody-associated syndromes have been an object of intense research. They result from the immune response against ion channels (e.g., leucine-rich glioma inactivated-1 protein (LGI-1) and contactin-associated protein 2 (CASPR2)) that are complexed with voltage-gated potassium channels (VGKC), voltage-gated calcium channels (VGCC), or neuronal receptors (e.g., NMDA, AMPA, GABA, and mGluR) [16]. These entities have been recognized as a separate clinical problem in which antibody-mediated responses are perceived as a prevalent pathogenic mechanism. This is consistent with the fact that immunomodulatory treatments are often effective in these patients, and this is reflected in clear therapeutic guidelines [17]. In an animal model, the administration of anti-mGluR1 antibodies into the subarachnoid space induced severe ataxia as a short-term effect [10]. Most of the neuronal surface antibody-associated syndromes are not related to malignancy and respond to immune therapy [18]. Anti-NMDA encephalitis has been reported to be associated with ovarian teratomas [19]. Clinical improvement in these patients has been observed along with a simultaneous decrease in antibody titers [20]. It has been demonstrated, however, that in paraneoplastic limbic encephalitis that is associated with neuronal surface antibodies, the intraneuronal antibodies often coexist, and the response to immunotherapy is poor [21]. Altogether, it seems that neuronal surface antibody-associated syndromes are a distinct group of disorders with an established pathogenic role of antibodies.

However, there have been studies that have not confirmed the causal relationship between onconeural antibodies and neuronal degeneration in the majority of PNS. The coculture of neurons with the anti-Hu and anti-Yo antibodies did not trigger cell death, but it induced the expression of adhesion molecules and more intense nerve cell differentiation [22]. Thus, one may hypothesize that onconeural antibodies influence the functional status of neurons and do not necessarily lead to cell death. Moreover, studies on the passive transfer of antibodies that were accompanied by complement or mononuclear cells [23] or the intracerebral injection of antibodies did not cause cell loss [23, 24]. The immunization of mice with either Hu [9, 25] or Yo [26] antigens induced only antibody production in the absence of neurologic pathology. Pathological studies of nervous tissue have revealed either no [27] or little [28] complement deposits, which is further evidence against isolated antibody-mediated cytotoxicity. It should be considered that blood-brain barrier breakdown might be a prerequisite for antibody-mediated toxicity. Indeed, the disruption of this barrier has been described in the course of an animal model of paraneoplastic cerebellar degeneration (PCD) [29]. Taken together, but excluding some surface antibody-associated syndromes, it seems that antibodies take part in the pathogenesis of PNS as one factor among others, but they alone are not a sufficient condition to induce the PNS. Hence, it is worth explaining how cell-mediated immune responses could contribute to the emergence of PNS.

### 2.1. Nervous System Infiltrates

**Figure 1:**
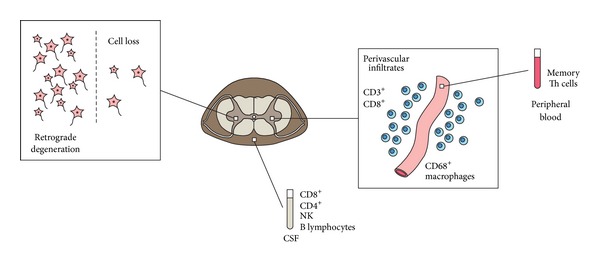
Lymphocytes of various subpopulations are found in affected regions of the nervous system, cerebrospinal fluid, and blood. The regions of the infiltrates do not always coincide with the area of cell loss that presumably comes from the retrograde degeneration.

Mononuclear infiltrates are observed in the affected regions of the nervous system and correspond to the clinical signs and symptoms in the course of PNS [13, 30]. Thus, in PEM that is associated with sensory neuronopathy, infiltrates were found predominantly in the hippocampus, medulla, cortex, spinal cord, and dorsal root ganglia [27]. They were localized both in the perivascular space and in the parenchyma [31]. In PCD, the degeneration of Purkinje cells in the cortex is found, but the infiltrate itself is often present either within the dentate nucleus [32, 33] or in the cerebellar white matter and pons [30]. It is hypothesized that the cell body or axonal damage in the adjacent area triggers, as a secondary process, neuronal loss in the cortex as a result of a retrograde degeneration (Figure 1). The transfer of T lymphocytes from experimental animals that were previously immunized with paraneoplastic neurological Ma protein (PNMA1) antigen from another mouse triggered infiltration into brain tissue [34].

In immunohistopathological studies, the infiltrates consist mainly of CD3^+^ and CD8^+^ lymphocytes [32, 35] whereas CD4^+^ cells are less numerous [14], indicating predominance of cytotoxic T lymphocytes (CTLs) over T helper (Th) cells. The plasma cells and B lymphocytes, however, have also been identified at an early stage of PNS to be related to anti-Ma2 antibodies [36, 37]. An increased population of macrophages (CD68^+^) has also been detected in paraneoplastic ganglionitis [38].

Regions of lymphocytic infiltration highly express intercellular adhesion molecule 1 (ICAM-1), which favors immune cell diapedesis [27]. Within infiltrates in the nervous system, no expression of MHC class I molecules has been identified. This implies some other mechanisms of cell death than those mediated by MHC class I restricted cytotoxic T cells [13]. Such a phenomenon supports the idea of the cross-presentation of neuronal antigens on the tumor cells, which coexpress class I MHC antigens and thus enable potentially excessive lymphocyte activation. Indeed, although all small-cell lung cancer (SCLC) and the majority of neuroblastoma tumors express HuD antigen, the onconeural antibodies are found far more frequently if the tumor cells express class I MHC at the same time [33, 39]. The increasing extent of this antitumor response may lead to the destruction of the nervous tissue that shares the same antigen profile [40].

### 2.2. Lymphocytes in Peripheral Blood and Cerebrospinal Fluid

Pleocytosis, along with increased protein concentrations and oligoclonal bands, appears often in the course of PNS [41, 42]. Normal results of cerebrospinal fluid (CSF) analyses were found in only 7% of the PNS patients. Increased cell counts are especially present at early stages of the disease during the first 3 months after the onset of neurological symptoms [42]. Thus, it can be hypothesized that, at the very beginning, PNS have an inflammatory course that subsequently turns into a noninflammatory or neurodegenerative phase. In patients with PCD, lymphocytes constitute the majority of cells that are found in the CSF [43]. Their identification presents a similar profile to the populations that are found in the tissues. The counts of T cells, CD8^+^ cells, CD4^+^ cells, natural killer (NK) cells, and, especially, B cells are increased, whereas natural killer T (NKT) cells are relatively decreased in the CSF in PNS patients when comparing cancer and noncancer patients [44] (Figure 1). The increased B cell count explains the phenomenon of intrathecal antibody production. Indeed, onconeural antibodies are present in the CSF in the majority of patients, and, most often, their titer is higher than in serum [3]. Interestingly, patients with anti-Hu syndrome and high pleocytosis have a better overall survival rate [42].

The analysis of peripheral blood has revealed increased memory Th cells (CD45RO^+^CD4^+^) [45] and decreased NKT cells [44]. The suppressive function of NKT cells is raised in the naturally ineffective tumor immunity [46]. Their influence, however, is ambiguous as they have been reported to exert both inhibitory and antitumor promoting activities [47].

### 2.3. The Specificity of Lymphocyte Reactions

The increased number of particular cell types does not answer the question of whether these are the same lymphocytes that react against both a tumor tissue and a nervous tissue. The tumor-infiltrating lymphocytes have been shown to react specifically with bioethylated HuD antigen [48]. It appears that peripheral blood lymphocytes are also active against the same antigens as the antibodies that are used in diagnostic tests. Specific CTLs have been found in both acute and chronic phases of PCD [43] and to coexist with ovarian [49] and breast tumors [50]. The reactivity of these cells was restricted to MHC class I signaling. Their existence was not so evident in Hu syndrome that is associated with SCLC. Peripheral blood mononuclear cells [51] and, specifically, CD8 T lymphocytes [52] that are aggressive towards Hu antigen-presenting fibroblasts have been detected only in a few cases. T lymphocytes that are derived from healthy subjects may be potentially active during stimulation in the presence of HuD-derived epitopes [53]. Similarly, in a study with a normal murine T cell repertoire, the cytotoxic CD8 lymphocytes were reactive against HuD antigen, but only after *ex vivo* stimulation [54]. The HuD immunogenicity that is accompanied by inhibited cell-mediated reactions resulting in antigen tolerance explains why PNS develops only in some patients with SCLC, although HuD antigen is expressed by all tumors. The presence of antigen-specific CTL in the course of Hu syndrome has only been partially confirmed by another study [55]. The incubation of peripheral blood lymphocytes with HuD antigen led to the proliferation of memory Th cells of the Th1 subtype (CD45RO^+^CD4^+^) [45]. CSF analysis has not revealed lymphocytes that are sensitive to HuD antigen in PNS patients with anti-Hu antibodies [56]. Different SCLC patients with anti-Hu antibodies did not share the same CD8 T lymphocyte responses, which may involve the following:

1. typical cytotoxic T lymphocytes producing IFN-*γ* or
2. atypical CD8^+^ T cells secreting IL-13 and IL-5, without cytotoxic activity, that are missed in standard functional assays [57].

Such a distinct modulation of immune response could be responsible for the development of PNS symptoms in certain patients, even though the HuD antigen is expressed in almost all SCLC tumors. This discrepancy between the frequency of onconeural antigen presentation in neoplasms and PNS incidence can also be partially explained by the genetic polymorphisms in the HLA system. It appears that HLA-DQ2 and HLA-DR3 alleles are more common in patients with Hu syndrome [58]. These HLA types have also been reported to be associated with autoimmune diseases, such as Graves-Basedow disease, myasthenia gravis, Addison's disease, and celiac disease [59]. In patients with PCD that is associated with the anti-Yo antibody, a high frequency of HLA A24 has been demonstrated [60, 61]. Strong associations between nonparaneoplastic Lambert-Eaton myasthenic syndrome and the HLA-B8, HLA-DQ2, and -DR3 types have been found [62]. The presence of HLA-B8 was related to a decreased incidence of SCLC, even among the population at risk, smokers. When SCLC patients developed LEMS, HLA-B8 positivity was associated with prolonged survival. This relationship between particular HLA types and the incidence of paraneoplastic reaction also supported the view that cellular immune responses have an impact on PNS emergence.

**Figure 2:**
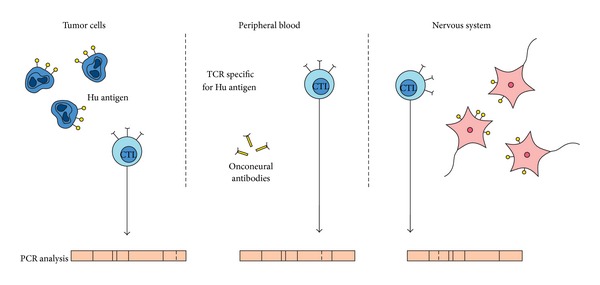
The same Hu antigen is present in both nervous tissue and tumor tissue. Cytotoxic T lymphocytes (CTLs) recognizing it are found in tumors and in the peripheral blood and nervous system. The clones of the TCR receptor appear to be similar in all three locations (based on the findings reported in [35, 63]).

It is noteworthy that the lymphocytes that are detected in peripheral blood, tumors, and affected nervous tissue originate probably from the same clone population. Based on an analysis of the TCR receptor gene [35] in Hu syndrome patients, the lymphocyte populations of similar clones have been identified. There were clones found in both blood and nervous tissue but not in lymph nodes and in another case in dorsal root ganglia and nodes, but they were absent in blood at the same time. The spectratyping of complementary-determining region 3 (CDR3) of the TCR receptor that was performed in a patient with anti-Hu encephalitis in the course of adrenal neuroblastoma [63] has shown a narrower repertoire of TCR in the CSF compared to the polyclonal spectrum in peripheral blood or tumor tissue. Even if TCR clones were different, some of them appeared functionally identical. Thus, probably, immune reactions that were initiated in one location were subsequently effective in different tissues (Figure 2).

### 2.4. Lymphocyte Function

The CTLs induce cell death that is mediated by at least three different mechanisms [32, 64] involving the following:

1. tumor necrosis factor (TNF) receptor,
2. FasL receptor activation,
3. granule excretion that destroys cells by the effects of granzyme-B, perforin, and T-cell restricted intracellular antigen-1 (TIA-1).

The first two trigger a cell to launch apoptosis through the caspase pathway. Granule excretions kill cells either by mitochondria permeabilization or through caspase processing [65]. However, the markers of the apoptotic pathway (FasL, Bcl-2, and caspase-3) are not present in infiltrates in contrast to the excessive expression of TIA-1 molecule [32] and granzyme B [36].

**Figure 3:**
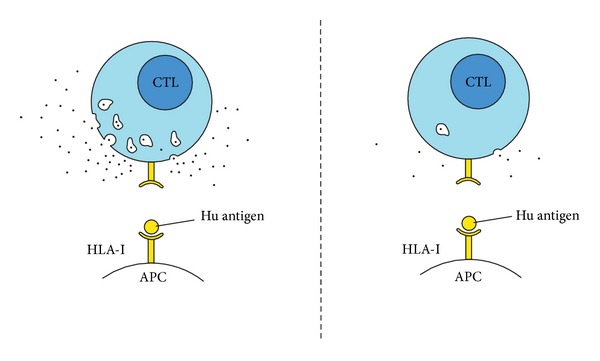
Cytotoxic T lymphocytes that are derived from PNS patients appear to be more aggressive towards cells presenting the Hu antigen.

Functional studies have shown that T lymphocytes are more active against particular antigens in the PNS [45, 55]. Lymphocytes that are isolated from patients with anti-Hu syndrome are more reactive against 13 out of 19 selected sequences of the antigen (Figure 3), whereas controls exhibit such a response against only 3 epitopes only temporarily. It was more intense in patients whose PNS were diagnosed less than 3 months before. One year after the diagnosis of PNS, the reactivity was significantly lower [55]. The lymphocytic activation coexisted with the clinical presentation of Hu syndrome. In contrast, in asymptomatic periods, such cell activity was decreased. These findings support the idea of cellular responses that are involved in the pathogenesis of PNS. *In vitro* studies have also confirmed the sequential nature of the cytotoxic response. The activity of cytotoxic lymphocytes against the recombinant Yo antigen and autologous dendritic cells has already been found after 4 days of incubation, but it increased significantly after 2 months of culture [49].

The particular lymphocyte subtypes differ in activity when in contact with onconeural antigens. An *in vitro* stimulation with HuD antigens has been shown to trigger the proliferation of memory T cells [45] and increase the interferon-*γ*/interleukin-4 ratio, which indicates an escalation of cell-mediated responses that are typical for the Th1 subpopulation rather than a humoral one.

In contrast, regulatory T lymphocytes (T_reg_) are less active in PNS patients [66] as is reflected by the significantly decreased expression of mRNA for FOXP3, TGF-*β*, and CTLA4, indicating their dysfunction.

## 3. PNS-Associated Neoplasms and Antitumor Responses in PNS

The PNS diagnosis is an indication for the search of an underlying neoplastic disease [7, 67]. The algorithm of the malignancy diagnosis in PNS, even if based on the guidelines [68], however, remains a clinical challenge. It comes mainly from the small-sized tumors that affect this group of patients. Hysterectomies that are performed in Yo-syndrome patients have shown that the tumors of female genital organs are detectable solely during a laparotomy [69] or microscopically [70]. The neoplastic disease is frequently limited and its course is accompanied by either none or small-sized metastases, in contrast to the much more severe clinical forms in patients without paraneoplasia [67, 69]. The frequency of PNS in lung cancer patients may reach 30%, and the presence of well-defined onconeural antibodies has been observed in 20% [71]. The localization of the SCLC tumors is often limited to the chest with the involvement of small mediastinal lymph nodes, without any distant metastases [3]. Moreover, a few cases of spontaneous tumor regression have also been reported [6, 72]. Nevertheless, sometimes a poor survival in these patients results from the severe course of the neurological syndrome itself [3, 67]. The PNS-associated tumors do not differ histologically from those found in the general population [73].

Onconeural antigens are found in tumor cells. They are detected in many neoplasms, even though they are not accompanied by a paraneoplastic reaction [74, 75]. Amphiphysin antigen is expressed in breast tissue [75], and Cdr2 is expressed in normal ovarian tissue [76]. This explains why humoral immune responses against normal ovarian tissue can be observed in patients with ovarian tumors [77]. In contrast, HuD antigen that is expressed by all SCLC cells is found exclusively in the nervous system in normal conditions [78]. Though onconeural antigens are expressed in a wide spectrum of tumors, PNS develops only in some patients. Most of the onconeural antigens are localized intracellularly in neurons [79], and this is another argument that underscores the importance of cell-mediated responses. In tumor cells, however, the HuD antigen is found equally at the cell surface, which facilitates its presentation to APC [80]. Hu is an RNA-binding protein [81], and it probably plays a role in posttranslational modifications [48, 82]. Cdr2 protein binds to c-Myc transcription factor, which is an oncoprotein, and downregulates its activity [83]. An interaction of the Cdr2 antigen and anti-Yo antibodies may lead to the upregulation of c-Myc and, consequently, to neuronal apoptosis. Thus, it can be hypothesized that small-scale antibody-mediated apoptosis leads to the facilitated antigen presentation to APC cells that subsequently promotes a secondary and more intense cell-mediated response. The Cdr2 antigen also has a leucine-zipper motif, and, thus, it potentially functions as a transcription factor that interacts with DNA [84, 85]. The reaction directed against this protein may interfere with gene expression, in both neurons and tumor cells [23]. In breast cancer patients with anti-Yo antibodies, which bind to the Cdr2 antigen, an overexpression of human epidermal growth factor receptor 2 (HER2) has been found compared to anti-Ri subjects. This may suggest a possible role of HER2 in PNS pathology [86]. Altogether, the most common onconeural antigens, Hu and Cdr2 proteins, have been demonstrated to take part in the regulation of gene expression and proliferation. Thus, an interaction between antibodies and those proteins may lead to the disturbed function of tumor cells.

As PNS are rare disorders, there is a methodological limitation in performing case-control studies on the role of the immune response in tumor evolution. However, reports are available which demonstrate that, in patients with PNS, the neurological symptoms and survival vary with both the type of associated onconeural antibody and the type of tumor [87]. The tumors underlying PNS reveal hallmarks of immune antitumor responses. First, high contents of onconeural antibodies are found in the tumor area [78]. Moreover, the antibodies are detected within tumor cells. Their concentration, however, is proportional to the levels in serum [30]. However, *in vitro* studies have not confirmed the toxic role of antibody deposits that are found within the nucleus of tumor cells [88]. Nevertheless, high serum titers of onconeural antibodies have been associated with slower tumor progression and better survival in patients with SCLC and PNS [5]. All patients in whom malignancies were not detected had high antibody concentrations [89]. Interestingly, among the patients with SCLC, individuals with anti-CV2/CRMP5 antibodies had better overall survival compared to the individuals with anti-Hu antibodies [87]. Secondly, in the course of Hu syndrome, the infiltrates were revealed in the SCLC tumor tissue [32]. They were mainly composed of CD8 lymphocytes. The intense plasma cells infiltrates have been found in ovarian tumors in PCD [30, 41, 89]. In some of these patients, no metastases were found [30]. Nonetheless, there were patients with few lymphocytes in tumor tissue. This coincided with almost complete Purkinje cell loss without immune infiltration in the cerebellum. In patients without cerebellar and tumor infiltrates, the course of the neoplastic disease was more severe [30]. Autopsies have revealed disseminated and distant metastases. It can be hypothesized that, initially, the majority of onconeural antigens had been destroyed, and an intense immune stimulation could no longer be maintained. It is possible also that the immunosuppressive influence that was exerted by the tumor inhibited the immune reaction at that moment. The experimental model that mimics the antitumor response in PNS is consistent with the clinical observations. Immunization with the Hu-antigen DNA plasmid induced tumor growth inhibition or even its rejection in mice. The infiltrate within the tumor tissue contained predominant CD3^+^ lymphocytes and a high CD8^+^ : CD4^+^ ratio [25]. The phenomenon of the antitumor response in PNS requires, however, further studies. As paraneoplastic disorders are relatively rare, it is difficult to draw decisive conclusions about the beneficial effects of immune response on the tumor evolution.

An effective antitumor immune response is also probable in patients with onconeural antibodies who do not manifest neurological symptoms. SCLC patients with low titers of anti-Hu antibodies more often had tumors that were limited to lung and mediastinum [90–92]. Their survival, and their response to therapy were better [90, 91]. Anti-Hu serum is more toxic for human tumor cell lines than the serum that is derived from SCLC patients without antibodies. Surprisingly, its toxicity is not mediated by anti-Hu antibodies. Moreover, the same toxic effects of anti-Hu sera were observed on both cells expressing Hu and cells without this antigen. The cytotoxic effects of TNF-*α* were also excluded [93]. Similarly, serum anti-Hu antibodies in neuroblastoma patients were associated with longer median survival [39]. Anti-Purkinje cells and anti-Ri antibodies have also been found in ovarian cancer patients without neurologic pathology [94, 95]. Their detection, however, was not associated with improved outcome [95]. Their prevalence was found especially in advanced stage ovarian carcinomas [96].

## 4. Discussion 

The suggested pathomechanisms that may link antitumor defense and cell-mediated immune response in PNS are based on the following:

- increased number and upregulated CTL activity as well as, at least temporarily, specificity against onconeural antigens that are shared by tumors and nervous tissue;
- less functional Treg cells in PNS that enable more aggressive antitumor responses;
- the natural restriction of onconeural antigens to immune privileged tissues, including the nervous system, that may decide more aggressive attacks mounted towards cells presenting them.

The clinicopathological observations suggest the sequential evolution of PNS. The presence of infiltrates in the nervous system, the cell activity, and specificity were more intense if the investigation was conducted in the first months of the disease. Positron emission tomography (PET) studies have revealed hypermetabolism of glucose in acute stages of the disorder that disappears in time [97]. It is possible that, as long as there are many cells in the nervous system presenting onconeural antigens, such a reaction is intense. In a subsequent phase, the antigen-stimulating response becomes scarce, which renders the immune system less aggressive, and the antitumor response becomes less effective. The gradual course of PNS may also come from the influence that is exerted by the tumor. The immunosuppressive cytokines or the activation of regulatory T cells may influence the repression of an initially intense immune attack. Once the antitumor response is less effective, the malignancy manifests clinically. This would be consistent with the fact that PNS often antedate cancer diagnoses. These observations emphasize the significance of the urgent management of PNS. It may reduce the severe neurologic implications and enable the detection of malignancy at a very early stage. That is why distinct guidelines for tumor screening have been introduced in patients with PNS [68]. Moreover, the recommended diagnostic procedures in patients with epilepsy, psychosis, and dementia that may result from autoimmune encephalopathy as a remote effect of cancer also include the evaluation of onconeural or neuronal surface antibodies [17].

The function of T regulatory cells should be further studied, including the previously described expression of CTLA-4 and FOXP3. It has already been shown that their expression is diminished [66]. However, it is not clear whether such a status is stable and whether it coincides with the emergence of a tumor. Another important issue is the clinical application of T_reg_ cells in the treatment of PNS. Such a strategy is currently under study in other autoimmune diseases. The transfer of T_reg_ cells that are multiplied *ex vivo*, FOXP3-transduced lymphocytes, or the infusion of CTLA-4 like antibodies could appear efficient.

However, the cell-mediated reaction is not always involved in the neuronal damage in PNS. Pathological studies have sometimes revealed significant cell loss without infiltrates [41]. Interestingly, in these patients, there are scarce inflammatory infiltrates in the tumor tissue as well [30]. This suggests that, at the moment of autopsy, all of the cells presenting onconeural antigens were already eliminated. One should also consider that the paraneoplastic degeneration is mediated by a different noninflammatory mechanism. An immune response may appear as a result of a secondary reaction to neuronal apoptosis that is induced by a different factor. There is basis to suspect that paraneoplastic syndromes emerge as a result of metabolic disturbances that are triggered by malignancy. There are a number of patients with PCD without any antibodies [98]. PET studies have demonstrated impaired glucose metabolism in nervous tissue in PNS [97, 99]. In an experimental model of neoplastic disease, cerebellar degeneration with Purkinje cell loss may develop without onconeural antibodies, but the involvement of chemokines and blood-brain barrier disruption was observed [29].

To conclude, the humoral response is an established biomarker in the diagnostic procedures in cases of suspected PNS. It can be promising, however, to use laboratory tests of cellular immune responses in routine diagnosis and monitoring of patients undergoing remote effects of malignancy on the nervous system.

## Figures and Tables

**Table 1:** The most common paraneoplastic neurological syndromes and associated tumors [1, 17, 100].

Typical paraneoplastic syndrome	Tumor
Limbic encephalitis	Small-cell lung cancer
	Thymoma
	Ovarian teratoma
	Hodgkin lymphoma
	Testicular seminoma

Paraneoplastic cerebellar degeneration	Ovarian cancer
	Breast cancer
	Small-cell lung cancer
	Thymoma
	Hodgkin lymphoma

Subacute sensory neuropathy	Breast cancer
	Small-cell lung cancer
	Thymoma

Opsoclonus/myoclonus syndrome	Neuroblastoma (children)
	Small-cell lung cancer
	Breast cancer
	Testicular seminoma

Lambert-Eaton myasthenic syndrome	Small-cell lung cancer
	Thymoma

**Table 2:** The most common onconeural antibodies and superficial antigen antibodies [1, 17, 100].

Type of antibody	Antibody	Tumor
Well-defined onconeural antibodies	Anti-Hu	Small-cell lung cancer
	Anti-Yo	Ovarian cancer
		Breast cancer
	Anti-Ri	Small-cell lung cancer
		Breast cancer
	Anti-CV2	Small-cell lung cancer
		Thymoma
	Anti-Ma/Ta	Testicular seminoma
		Small-cell lung cancer
	Antiamphiphysin	Breast cancer
		Small-cell lung cancer

Antibodies against superficial antigens	Anti-NMDA	Ovarian teratoma
		Testicular teratoma
		Small-cell lung cancer
	Anti-AMPA	Thymoma
		Small-cell lung cancer
		Non-small-cell lung cancer
	Anti-GABA	Small-cell lung cancer
	Anti-LGI1	Small-cell lung cancer
	Anti-CASPR	Thymoma
		Small-cell lung cancer
		Hodgkin lymphoma
	Anti-VGCC	Small-cell lung cancer
